# Different clinical allergological features of *Taenia solium* infestation

**DOI:** 10.1186/s12948-016-0056-x

**Published:** 2016-12-07

**Authors:** Paola Lucia Minciullo, Antonio Cascio, Stefania Isola, Sebastiano Gangemi

**Affiliations:** 1 School and Division of Allergy and Clinical Immunology, Department of Clinical and Experimental Medicine, University Hospital “G. Martino”, Messina, Italy; 2 Department of Health Promotion Sciences and Mother and Child Care “G. D’Alessandro”, University of Palermo, Palermo, Italy; 3 Institute of Applied Sciences and Intelligent Systems (ISASI), Messina Unit, Messina, Italy

**Keywords:** Allergy, Anaphylaxis, Angioedema, Asthma, Cysticercosis, Urticaria, *Taenia solium*

## Abstract

The tapeworm *Taenia* (*T.*) *solium* can be responsible for two different conditions: taeniasis and cysticercosis. Helminth infections in human host cause an immune response associated with elevated levels of IgE, tissue eosinophilia and mastocytosis, and with the presence of CD4+ T cells that preferentially produce IL-4, IL-5, and IL-13. Individuals exposed to helminth infections may have allergic inflammatory responses to parasites and parasite antigens. PubMed search of human cases of allergic reactions occurring during *T. solium* infestation was performed combining the terms (allergy, urticaria, angioedema, asthma, anaphylaxis) with *T. solium*. A study was considered eligible for inclusion in the review if it reported data on patients with *T. solium* infestation who had signs or symptoms of allergy. In literature we found six articles reporting the association between an allergic reaction and *T. solium* infestation: two cases of urticaria, two cases of relapsing angioedema, one case of asthma and two cases of anaphylaxis. Despite the large diffusion of *T. solium* infestation, we found only a few cases of concomitant allergic reaction and the presence of Taenia in the host. The association between *T. solium* infestation and allergic manifestations has never been clearly demonstrated, and in absence of a well-documented causality the hypotheses are merely speculative. Therefore, the association between Taenia infection and allergy needs to be thoroughly studied to better clarify if this association may really exist and which is the pathogenetic mechanism supported.

## Background

The tapeworm *Taenia* (*T.*) *solium* can be responsible for two different conditions: taeniasis and cysticercosis. Taeniasis is infection with an adult tapeworm, while cysticercosis is infection with larval stages (of *T. solium*) in body tissues. Taeniasis occurs only in the human host, after ingestion of undercooked pork infected with cysticerci. In taeniasis an adult worm is present in the intestine, and the infestation is usually asymptomatic and generally recognized when segments of proglottids are found in stool specimens. Cysticercosis is caused by ingestion of food contaminated with feces, or by autoinfection. In the latter case, a human infected with adult *T. solium* can ingest eggs produced by that tapeworm, either through fecal contamination or, possibly, from proglottids carried into the stomach by reverse peristalsis. Once eggs are ingested, oncospheres hatch in the intestine invade the intestinal wall, and migrate to striated muscles, as well as the brain, liver, and other tissues, where they develop into cysticerci. Cysticercus consists of a fluid-filled bladder elongate and oval, measuring 5–15 mm with a single protoscolex [1–3]. Cysticercosis of the human nervous system, called neurocysticercosis, may cause different clinical manifestations based on the characteristics of the parasites and the inflammatory response of the host. The intraparenchymal neurocysticercosis mainly causes seizures, whereas the subarachnoid infection with parasite infiltration cause hydrocephalus that may be fatal [4].

Diagnosis of cysticercosis is based on a combination of clinical presentation, imaging findings, history of exposure, and serologic testing as the detection of IgG antibodies to *T. solium* using an ELISA technique. In neurocysticercosis the neuroimaging techniques are considered the gold standard for the diagnosis. However, the detection of specific antibodies against *T. solium* cysticercal antigens in cerebrospinal fluid may be a useful adjunctive diagnostic test. A high rate of patients with neurocysticercosis presents specific IgE and IgG (especially IgG4) that is indicative of intrathecal antibody production [5].

The human immune response to helminth infections is associated with several immunomodulatory mechanisms that can have different and sometime opposite consequences. Asymptomatic carriers often present immunologic hyporesponsiveness and altered cytokine profiles favoring Interleukin (IL)-4 over inflammatory mediators such as IFN-γ and with a prominent role played by IL-10 and TGF-β. On the contrary, patients who develop a symptomatic disease, present loss of the immune tolerance with predominance of Th1 and Th17 responses.

Dendritic cell antigen presentation, B cell antibody production, and epithelial cell alarmin release are other pathways of immune activation suppressed during helminth infection. On the contrary, T regulatory (Treg) and Breg cell differentiation is induced.

Thus, the immunomodulation caused by helminth infection can be useful for the host in protecting from allergic, autoimmune and inflammatory diseases, but, at the same time could be dangerous in reducing tumor immunosurveillance, depending on the characteristics of the parasite, the clinical course of the subject and the chronicity of infection [6].

The inverse association between helminth infection and allergy, first postulated by Bell in 1996 [7] is, today, largely assumed. The global increase in allergy especially in urban areas has led researchers to propose a modified hygiene hypothesis in which the decline in helminth infections is associated with an increase in allergic diseases [8].

However, very little data are available to explain how helminth infection might protect against allergy.

Allergy and helminths induce strongly Th2-skewed responses associated with cytokines such as IL-4, IL-5, and IL-13, with mastocytosis, eosinophilia, and antibody class-switching to produce IgE. However, the two conditions are completely different. Allergy occurs in predisposed people and is due to a polygenic disorder associated with Th2 responses and IgE expression, whereas the Th2 activation during helminth infection is a normal physiological reaction. Moreover, this response is moderated by helminths with the induction of Treg and Breg cells and the production of immunoregulatory cytokines and IgG4 that contrast IgE [9].

However, some helminth infections, especially by *Ascaris lumbricoides* and *Anisakis simplex* are associated with an increased incidence of allergic diseases, such as asthma, allergic rhinoconjunctivitis, atopic dermatitis, chronic urticaria, until to anaphylaxis [10–14]. This is probably due to a cross-reactivity between helminth proteins, as tropomyosin, and specific allergens, especially in *A. lumbricoides* infection [9, 15].

The aim of our study was to depict the different clinical allergological features of *T. solium* infestation.

## Literature review

PubMed search of human cases of allergic reactions occurring during *T. solium* infestation was performed combining the terms (allergy, urticaria, angioedema, asthma, anaphylaxis) with *T. solium*. A study was considered eligible for inclusion in the review if it reported data on patients with *T. solium* infestation who had signs or symptoms of allergy.

## Results

In literature we found six articles reporting the association between an allergic reaction and *T. solium* infestation: two cases of urticaria, two cases of relapsing angioedema, one case of asthma and two cases of anaphylaxis (Table 1).

**Table 1:** Different allergic manifestation associated to *Taenia solium* infestation

Allergic manifestation	Sex	Age (years)	Nationality	Reference
Urticaria	Female	44	Indian	Gupta et al. [16]
Urticarial vasculitis	Male	36	Ecuadorian	Shaigany et al. [17]
Angioedema	Female	2	Italian	Accomando et al. [18]
	Male	4	Italian	
Asthma	Female	35	Mexican	Rodríguez Orozco [19]
Anaphylaxis	Male	20	Indian	Singh et al. [20]
	Male	26	Italian	Minciullo et al. [1]

### Urticaria

Gupta et al. [16] described a case of a 44-year-old woman with a history of urticaria of 1 month duration. No relevant causes of urticaria were established. The patient was treated with antihistamines with a partial response. Subsequently, she presented a painful swelling over right cheek treated with antibiotics without resolution. The patient presented also mild eosinophilia. Ultrasonography on the swelling region revealed the presence of intramuscular cysticercosis with surrounding phlegmon. The woman was treated with albendazole 15 mg/kg for 1 month, initially associated to steroids to reduce inflammatory response. After treatment the Authors observed a reduction in the size of the swelling with no more evidence of active cysticercosis in the muscle and also the resolution of urticaria.

A case of urticarial vasculitis associated to neurocysticercosis has been reported [17]. A 36-year-old man presented afebrile seizure and a 3- to 4-month history of intermittent pruritic widespread rash associated with a burning sensation. He had edematous plaques and scattered erythematous papules on both posterior thighs. Skin biopsy showed leukocytoclastic vasculitis, leading to a diagnosis of urticarial vasculitis. The patient presented a cystic lesion in his brain with degenerating cysticercus. Despite the serum antibody testing for cysticercosis was negative and cysticercosis immunoglobulin (Ig)G antibodies had undetectable levels in cerebrospinal fluid, the clinical feature, the exam of the cystic lesion and the MRI images fulfilled the criteria of neurocysticercosis. The patient was treated with albendazole 400 mg twice daily and his urticarial vasculitis resolved within a few days.

### Angioedema

Two cases of angioedema related to cysticercosis have been reported by the same Authors [18]. The patients, a 2-year-old female and a 4-year-old male, presented a history of relapsing and migrant angioedema in the upper and lower limbs and in the body trunk and the female patient in the genital region also. The angioedema was associated to prurigo and rash. Blood and stool parasitological exams were normal except for hypereosinophilia; skin prick test for the most common inhalant and food allergens was negative. Test for cysticercosis showed high positivity, however, no presence of *T. solium* cyst was found. Treatment with albendazole 5 mg/kg every 8 h for 4 months led to normal levels of eosinophils and to the disappearance of angioedema.

### Asthma

A case of asthma exacerbation related to neurocysticercosis has been reported by Rodriguez Orozco [19]. A 35-year-old female with a 2 years history of mild asthma presented headache, nausea, vomiting and an episode of convulsion in the last 3 months. The electroencephalogram and the neurological evaluation were normal. During the last 2 months the patient presented nocturnal dyspnea, wheezing and a worsening of headache and nausea and she needed hospitalization. She was treated with inhalant corticosteroids and beta-agonists, antileukotrienes and oral corticosteroids. Because of headache and nausea persisted, the patient underwent cerebral tomography that showed the presence of two parenchymal cysts. One was a colloidal cyst interpreted as cyst with a dying parasite, the other one as a dead cyst. The Author concluded that the neurological manifestations were linked to the parasite death. Since asthma exacerbation and neurological symptoms appearance were concomitant, the Author hypothesized that the two entities were related, however, it is difficult to establish in this case if neurocysticercosis is a concomitant event or a trigger factor for asthma exacerbation.

### Anaphylaxis

Two case of anaphylaxis related to *T. solium* infestation have been reported [1, 20]. The first was a 20-year-old man who presented with sudden onset of transient generalized urticarial rashes, hypogastric pain that spread to the whole of the abdomen, vomiting, watery diarrhoea and fever of 6 h duration. He also exhibited tachycardia, hypotension, diffuse abdominal tenderness, and rigidity. A diagnosis of appendicular perforation with pelvic collection was made and exploratory laparotomy was normal except for the presence of colourless, odourless gelatinous material in the pelvis. The appendix was histopathologically normal and the histopathological examination of this fluid showed cysticercosis presence. The patient was treated with albendazole 15 mg/kg for 28 days and no other signs of Taenia infestation were present in the follow-up.

The Authors hypothesized a rupture of a cyst in the peritoneal cavity that led to the anaphylactic reaction and the gelatinous content of the cyst caused a peritoneal inflammation mimicking the secondary peritonitis of appendicular perforation [20].

A case of fatal anaphylaxis has been reported by our group [1]. A 26-year-old male died during sleep and death did not seem linked to any previous known diseases. The cadaveric and histologic examinations and the necroscopy concluded that the cause of death was anaphylactic reaction. Specifically, swelling of the larynx, mucous plugging in the airways, pulmonary emphysema, visceral congestion, and splenic eosinophilia supported an allergic reaction. Moreover, in the intestinal loops several meters of a tapeworm with a long series of proglottids were found the worm was identified as *T. solium*.

The most common causes of anaphylaxis (drugs, foods, insect sting) were excluded by interviews with the man’s relatives, external examination of the body, and cadaveric examination.

The Authors hypothesized an autoinfection of larval forms by ingestion of eggs present on the man’s dirty hands or by reverse peristalsis had occurred and the larval forms developed into cysticercosal cysts. The rupture of a unknown cyst may have triggered the anaphylaxis. At the time of necroscopy, cysticercosal cysts were not searched in the examined tissues, but the presence of *T. solium* in the intestine, the pathologic findings at autopsy, and the absence of other possible causes of anaphylaxis make the association between anaphylaxis and cysticercosis plausible.

## Discussion

The association between allergic diseases, especially urticaria, and infectious diseases has been discussed for >100 years [11]. Several chronic persistent bacterial [21], viral [22], parasitic [10, 14] infections have been suspected to trigger allergic symptoms, ranging from urticaria to anaphylaxis.

*Taenia solium* infestation is endemic in countries in Sub-Saharan Africa, Latin America and Asia where conditions such as inadequate hygiene, poor sanitary conditions, open defecation, free roaming pigs and poverty permit the transmission of the disease [23]. However, three recent reviews indicate that autochthonous human *T. solium* taeniasis/cysticercosis may be possible in Europe [24–26], but current peer reviewed literature is biased towards Western Europe [27].

In this review we reported allergic manifestations related to Taenia infestation. Despite the large diffusion of *T. solium* infestation, we found only seven cases of concomitant allergic reaction and the presence of Taenia in the host. The clinical allergological manifestations reported in these cases are various, including urticaria, angioedema, asthma and anaphylaxis. Moreover, it is interesting to highlight that two cases are reported from India [16, 20], one from Mexico [19], one from USA but the patient was from Ecuador [17], and three from Italy [1, 18].

The association between *T. solium* infestation and allergic manifestations has never been clearly demonstrated, therefore, in absence of a well-documented causality the hypotheses are merely speculative. In some cases the connection between the infection and the allergic reaction is supported by the resolution of the allergic symptoms after surgery and albendazole treatment [16–18]; in other cases it is uncertain if there was just a coexistence of Taenia infection and allergy or if parasite infection was a trigger factor for allergic manifestations.

In any case, the rarity of the co-occurrence of the two entities remains unclear, since helminth infections are usually associated with low rate of allergies. However, in some infections (especially *A. lumbricoides* and *A. simplex*), allergic diseases are increased; this is sometime linked to a cross-reactivity between worm proteins and specific allergens, especially in *A. lumbricoides* infection [9, 15]. Nevertheless, this mechanism has not been observed in Taenia infection.

Therefore, the association between Taenia infection and allergy needs to be thoroughly studied to better clarify if this association may really exist and which is the pathogenetic mechanism supported.

## Abbreviations

*T.*: *Taenia*
Ig: immunoglobulin
IL: interleukin
IFN-γ: interferon-γ
Th: T helper
Treg: T regulatory cells
Breg: B regulatory cells
